# The urgency of addressing zoonotic diseases surveillance: Potential opportunities considering One Health approaches and common European Data Spaces

**DOI:** 10.1016/j.dib.2025.111332

**Published:** 2025-01-30

**Authors:** Nicola Riccetti, Serena Signorelli, Angela Fanelli, Emanuele Massaro, Manlio Bacco, Wojciech Szewczyk, Dolores Ibarreta, Juan Carlos Ciscar, Alessandro Cescatti, Sandra Coecke, Ilaria Capua

**Affiliations:** a European Commission, Joint Research Centre (JRC). Via E. Fermi 2749, 21027 Ispra Italy; b European Commission, Joint Research Centre (JRC). C. Inca Garcilaso 3, 41092 Sevilla Spain; c Johns Hopkins University SAIS Europe. Via B. Andreatta 3, 40126 Bologna Italy

**Keywords:** Epidemiology, Public health surveillance, Zoonoses, One Health, Disaster planning, Information dissemination, Data spaces, EHDS

## Abstract

Currently, transdisciplinary data from animal surveillance that are available for One Health approaches to public health are scarce, negatively impacting our ability to anticipate and prepare for future public health threats, particularly those involving zoonotic diseases with pandemic or epidemic potential. In this article, we explore the potential of the common European Data Spaces framework to enhance the availability of animal surveillance data, in order to better address public health threats. We propose building upon and expanding existing initiatives, such as the European Data Spaces for Health, Agriculture, and Green Deal, to design innovative services. These services could enable the integration of different data sources to inform research and policymaking on public health interventions. An overarching layer, populated with data and generating integrative information, could support a One Health approach to research and policymaking for the preparedness and anticipation of zoonotic diseases. Consequently, this approach might foster data sharing from Member States by leveraging existing developments within data spaces in terms of, for example, data security. It could also support researchers and developers in accessing transdisciplinary, stratified, and quality-controlled data for their projects.

## 1 Background

### 1.1 Zoonoses: definition and impact on public health

On March 11^th^, 2020, COVID-19 was responsible for more than 118,000 cases and 4,291 deaths when the World Health Organization (WHO) characterized the outbreak as a pandemic [1]. Since then, these figures have grown to more than 775 million cases and 7 million deaths worldwide, with an estimated cost of $13.8 trillion through 2024 [2], marking the COVID-19 pandemic as the most widespread and impactful zoonosis—defined as an infectious disease that passed from animals to humans [3]—of the last century [4]. However, by focusing solely on the figures of the COVID-19 pandemic, we risk overlooking other newly developed or resurging infectious diseases with epidemic or pandemic potential. Although not yet at the same magnitude as COVID-19, these diseases are entering our environment and affecting it at an unprecedented pace [5,6]. It would be of public health interest to consider these diseases collectively, as most of them share a common pathway [5]. While the European Union's position on the origins of SARS-CoV-2, the virus responsible for COVID-19, is not yet established, one of the main theories is that it resulted from a spillover event of a virus from animals to humans [7]. A *spillover* is defined as the process by which a virus is transferred from a known to a naïve host, in this case from an animal to the human population, through chance, novel or repeated exposure, or genomic changes [8]. Despite how random this process may seem, spillovers from animals represent a common pathway for diseases to emerge in humans [5]. Recent examples of spillover events that have led to epidemic diseases include SARS-CoV-2-closely related SARS-CoV, the virus responsible for SARS, and MERS-CoV, the virus responsible for MERS [9]. Both viruses are believed to have originated from bats, particularly horseshoe bats (genus *Rhinolophus*), with SARS-CoV being transmitted to humans from masked palm civets (*Paguma larvata*), and MERS-CoV from dromedary camels (*Camelus dromedarius*) [9]. Similarly, HIV and Ebola virus also resulted from spillover events from wildlife, with the former believed to have been transmitted to humans from chimpanzees (*Pan troglodytes*) [10]. Although wildlife is the predominant source, the spillover pathway from animals to humans is not limited to wild species. Several human diseases that have posed significant public health threats are the result of spillovers from domestic animals, such as influenza A, measles, rotavirus, smallpox, and tuberculosis [11].

Furthermore, while animals are capable of transmitting diseases to humans, they could also pose an additional threat through the development of secondary epidemiological cycles or different virus variants. An example of this might be the susceptibility of mustelids, especially farmed minks, to SARS-CoV-2 [12].

### 1.2 Limitations of veterinary surveillance for One Health approaches

For the reasons mentioned above, a One Health approach to surveillance and science for policy could be instrumental in enhancing both preparedness and anticipation of newly developed and resurging zoonotic diseases with pandemic or epidemic potential [13]. This is particularly relevant when acknowledging that anthropogenic environmental changes—such as loss of biodiversity, climate change, and overpopulation—are creating conditions conducive to zoonotic spillover events [14].

According to the One Health High-Level Expert Panel (OHHLEP), One Health is defined as “*an integrated, unifying approach that aims to sustainably balance and optimize the health of people, animals, and ecosystems*” [15]. The scientific community and public health policymakers have recognized the necessity of considering public health as a whole, encompassing humans, animals, and the environment. Similarly, the European Commission has developed a One Health agenda, and the five European Agencies representing the European Agencies Network on Scientific Advice (EU-ANSA) have recently published their joint One Health framework to support the implementation of this agenda [16]. Specifically, these agencies, which are the ones most closely related to One Health in scope—such as the European Centre for Disease Prevention and Control (ECDC), the European Chemicals Agency (ECHA), the European Environment Agency (EEA), the European Food Safety Authority (EFSA), and the European Medicines Agency (EMA)—aim, among other objectives, to enhance the scientific evidence base for One Health and to reinforce intersectoral coordination mechanisms [16].

However, these significant strides toward a more holistic approach to public health must be accompanied by acknowledgment of certain persistent limitations. While comprehensive datasets on animal diseases exist (e.g., Animal Disease Information System [ADIS]; World Animal Health Information System [WAHIS]), they are often based on infectious diseases that are reported by individual Member States according to European regulations on veterinary surveillance [17]. Nevertheless, the list of notifiable veterinary diseases is primarily focused on animal health priorities and does not necessarily include zoonotic diseases with pandemic or epidemic potential, which are more pertinent at the animal-human interface. For example, the WHO has developed a list of diseases considered major public health risks based on their epidemic potential and the availability of countermeasures. This list, known as the Blueprint list of priority diseases, includes COVID-19, Crimean-Congo haemorrhagic fever, Ebola and Marburg virus diseases, Lassa fever, MERS and SARS, Nipah and henipaviral diseases, Rift Valley fever, and Zika virus disease, as well as a yet-to-be-identified pathogen with the potential for serious international epidemics (listed as “Disease X”) [18]. However, only a few of these diseases are systematically included in structured data collection for surveillance, most notably COVID-19 and Rift Valley fever.

This shortage of data on zoonotic diseases with pandemic or epidemic potential may be further exacerbated when there is no standard case definition for these diseases, hindering the evaluation of pathogen circulation in specific areas. A recent example of this potential issue is the role of bovines as reservoirs for the influenza A virus subtype H5N1. Bovines are considered at risk of developing asymptomatic forms of the disease, potentially allowing undetected virus circulation if no serosurveillance program is in place. Given the increasing number of outbreaks in bovine dairy farms and the presence of cases in human dairy farm workers, there is a concern that, due to a lack of data collection and sharing, we may be losing track of the spread of a potential public health threat. Finally, to enhance the possibility of conducting more holistic research on risk assessment, additional information should be made available for research and development, such as livestock and farm density. However, this information might be sensitive for Member State Authorities to share publicly, especially if no privacy-preserving strategies are in place.

### 1.3 Common European Data Spaces

From our perspective, the framework of common European Data Spaces could offer a potential solution for some of these limitations. As part of the European strategy for data (2020), the European Commission has promoted the development of common European Data Spaces in several macro areas of public interest, including Manufacturing, Green Deal, Mobility, Finance, Energy, Agriculture, Public Administration, Skills, and Health [19].

These common Data Spaces are defined as “*decentralized infrastructures where diverse actors can share, access, and use data in a secure, reliable, and trustworthy manner, following common governance, organizational, regulatory, and technical mechanisms*” [19,20]. The European Health Data Space (EHDS) is an exemplary case among the 14 Common European Data Spaces currently under development, with strategic goals that include making data available to support prevention, detection, and treatment strategies, as well as to inform evidence-based decisions to improve the healthcare system [19,21]. The European Parliament recently approved the establishment of the EHDS, and the Regulation is expected to come into force at the end of 2024 and be applicable from 2026 [22]. However, the proposal for the Regulation of the EHDS does not explicitly mention animal health data, veterinary surveillance, or One Health surveillance—topics that could represent integrative layers of the EHDS, enhancing its potential for research, policymaking, and development [21].

In this article, we propose a One Health layer that is transversal to the EHDS, the Agricultural, and the Green Deal Data Spaces. Other initiatives may also be considered, such as the European Life Sciences Infrastructure (ELIXIR) and national nodes tasked with coordinating national data spaces for health, clinical, and epidemiological data [23]. By allowing the coexistence of information from different layers, rather than attempting to integrate them, the envisioned role of this transversal layer could be to expand the pre-existing Data Spaces to include One Health-related topics, with a special focus on the secondary use of health data to support health research, innovation, and policymaking [24,25]. The One Health layer has the potential to become a trusted ecosystem where data from professionals and authorities in the fields of health and agriculture can be brought together to design specific services that inform decision-makers, researchers, and citizens, as well as enhance economic and environmental performance across multiple domains.

The factual and economic justification for this layer might be represented by the increased promotion of public health at the EU level, a higher quota of shared data, and public health advantages in terms of preparedness, such as data-driven externalities between health subjects and the improved ability to harness information from the pooling of diseases that have a small footprint in individual Member States [25]. By their nature, the common European Data Spaces allow various actors, including private sector companies, public authorities, individual citizens, and researchers, to contribute to the data ecosystem. In our specific case, different stakeholders may find advantages in participating in this One Health layer. For example, public health authorities in Member States may be incentivised to facilitate the use of data by researchers and developers through a federated approach, standardizing procedures for data access and use, with built-in privacy safeguards to reduce the risk of data breaches. Researchers will benefit from reduced barriers in accessing transdisciplinary and potentially standardized data.

There are alternative approaches to the use of data spaces and a transversal layer on top of them, as we propose here. The first option is represented by a classical data sharing solution in which all data are centrally hosted, and another option could be the opposite solution, e.g., a fully decentralised system but with no common standards. Generally speaking, the situation can be described at present as a mix of those two approaches: a fair share of non-sensitive data is hosted in cloud-based solutions, and another share of data is in data islands; data exchange and use is hampered by the lack of commonly agreed data standards and procedures.

Considering pros and cons, a centralised solution offers an easy setup of the system but with a very limited control of the data by the owners; in fact, once in the central location, owners may struggle to regain full control of their data (in terms of update, delete, selective data access control policies). Focussing on data under discussion here, the narrow scope of the initiative could be a further obstacle to the setup of a potentially costly centralised solution. Considering instead the case of disconnected data islands, the predominant issues would be the governance framework and interoperability in a disconnected ecosystem; however, access control would be easier to implement for data providers because each of them hosts their own data and can allow or prevent access to them, enforcing control. The use of data spaces may unlock both pros, meaning that access control would stay fully in the hand of data providers/data owners, and the use of the dataspace protocol would ensure (at least minimal) interoperability. Finally, data of interest could be retrieved from the different sectorial data spaces without the need to set up an additional system, but only a software layer interconnecting the different spaces and using the same services each data offer (e.g., data catalogues, contracts, terms of use).

Major hindrances to the sharing of health data include privacy concerns and the risk of data leakages [25]. These issues could be minimised, as already proposed for the EHDS, by using anonymized or pseudonymised data, and decentralized data pools [25]. Furthermore, the integration of data from different sources, as data spaces aim to achieve, poses additional challenges such as limited data harmonization or lack of uniform coverage. While data spaces have the potential to mitigate some of these challenges, others remain outside their scope. For example, considering data exchanges, data spaces will utilize the data space protocol as a standard reference solution [26]. However, it must be noted that data coverage depends on data providers and the interworking of policies and procedures at national and EU levels, so uniform data coverage cannot be guaranteed by data spaces alone. Relevant data are collected by various entities across different locations, each with specific objectives and regulations to follow. The diversity of data providers ensures time- and location-dependent data, potentially enriching the information pool. Conversely, the data are likely collected according to different protocols and organised and stored in various formats, presenting challenges for semantic integration.

Data intermediation service providers and registered data altruism organizations—two entities regulated by Chapters III and IV of the Data Governance Act [21]—may play a key role in enabling citizens and companies (respectively considered as data subjects and data holders) to contribute to the data spaces through trusted organizations. For instance, farmers could monetize their One Health-related data (e.g., milk quality, livestock density) with the help of a data intermediary. Citizens could also participate by donating individually collected data (e.g., citizen science data on wildlife or infectious disease vectors) or data from smart appliances they own through a data altruism organization. A data intermediation service provider and a data altruism organisation could coexist in a data space, due to the different nature of these two entities. The former could act as an intermediator for more institutional entities (like farmers), to allow them to monetise their data, while the latter could act as a sort of an intermediary for less institutional actors (like citizens) that could contribute to the data space through the data they collect throughout their day-by-day life and that they decide to donate. The official recognition of both entities under the Data Governance Act could increase trust in the data space, increasing the participation of more data subjects and data holders. Furthermore, in the presence of trusted intermediaries in such an ecosystem, data collected from veterinary health personnel, which might otherwise fall outside of the obligation of notification, may contribute to new spontaneous data-sharing initiatives that provide additional insights for developing a One Health approach to surveillance.

The implementation of this One Health layer might therefore be transversal across several Data Spaces, leveraging ecosystems already under development and introducing novel services to mitigate and prevent public health threats, especially zoonotic diseases with pandemic or epidemic potential.

## 2 Summary

In this article, by highlighting the need for timely access to transdisciplinary data on animal diseases to facilitate surveillance for One Health research and policymaking, we aimed to explore the potential benefits of a One Health transversal layer within the common European Data Spaces framework. The current limitations in data sharing in this field restrict our ability to enhance preparedness for potential public health threats posed by zoonotic diseases with pandemic or epidemic potential. To support the implementation of the EU One Health agenda, high-quality and FAIR (Findable, Accessible, Interoperable, and Reusable) One Health-related data must be readily available to scientists, policymakers, and stakeholders.

In line with this, we discussed the potential advantages that the framework of common European Data Spaces could provide, especially in relation to the European Commission's One Health Agenda. Just as we advocate for a transdisciplinary approach to public health (i.e., One Health), we are also convinced that the ability to leverage data from various actively developing data spaces and to create novel services for prevention, detection, and treatment strategies will be instrumental for both research and policymaking. This will help in developing strategies to anticipate future health threats. More specifically, the data layer we proposed would be critical in supplying the necessary data for, for example, the most advanced applications of data-driven studies with AI, and for modelling the complex interplay between diseases, hosts, and the rapidly changing environmental conditions. As such, it would contribute to the advancement of Open Science in this scientific domain, which is poised to gain relevance under the challenging conditions posed by factors such as climate change. While advising for this development, we did not delve into the substantial efforts that such an initiative may require in terms of costs and the establishment of proper governance mechanisms. Nevertheless, we remain convinced of the paramount importance of data sharing in the public health domain, as demonstrated during the COVID-19 pandemic. Moreover, health preparedness and resilience, along with research and digital transition, are key pillars of the NextGenerationEU Recovery plan for Europe, which could therefore serve as a potential source of financial support. Additionally, the results of global data sharing efforts in pursuit of solutions to the COVID-19 pandemic may also assist in extending the reach of this federation of decentralized data ecosystems globally, contributing to a collective public health effort.

## Disclaimer

The views expressed in this article are purely those of the authors and may not, under any circumstances, be regarded as an official position of the European Commission.
